# “The Evidence I Need Is Change in a Child” Healthcare Provider Perspectives on Acupuncture Therapy for Children Diagnosed With Autism Spectrum Disorder (ASD): An Interpretive Description

**DOI:** 10.1177/27536130261448873

**Published:** 2026-05-05

**Authors:** Deborah Dong, Sunita Vohra, Hsing Jou, Sandy Thompson-Hodgetts

**Affiliations:** 1 Department of Paediatrics, Faculty of Medicine and Dentistry, 12357 University of Alberta, Edmonton, AB, Canada; 2 Department of Occupational Therapy, Faculty of Rehabilitation Medicine, 12357 University of Alberta, Edmonton, AB, Canada

**Keywords:** laser acupuncture, qualitative, healthcare views, children, autism

## Abstract

**Background:**

While parents of children and adolescents with autism spectrum disorder (autism) frequently seek complementary medicine to address health and functional concerns commonly experienced by their children, little is known about how conventional healthcare providers view acupuncture.

**Objective:**

To explore healthcare professionals’ (1) knowledge and views about acupuncture, including laser acupuncture, and (2) perceived reasons for recommending or not recommending acupuncture for children with autism.

**Design:**

This interpretive descriptive study involved semi-structured interviews. Purposeful sampling was used to recruit conventional healthcare providers who work with children with autism, including physicians and therapists. Data were coded and analyzed using an inductive thematic analysis approach.

**Results:**

13 healthcare providers were interviewed. Four themes were identified: (1) “Interesting, tell me more”: a lack of familiarity, but interest, in acupuncture for children with autism; (2) “I think we need to explore all avenues”: openness to integrative practices for a complex condition; (3) “The evidence I need is change in a child”: both empirical evidence and clinical experience are important to inform clinical decisions; and (4) Practical considerations to recommending acupuncture.

**Conclusions:**

Acupuncture is a modality that many professionals working with children with autism have not considered recommending due to lack of awareness and knowledge. Yet, the attitudes of our participants toward acupuncture as an adjunct therapy were generally positive. They were curious and open about exploring the evidence, and potential to meet gaps not currently met by conventional therapies. Laser acupuncture generated more interest than needle-based acupuncture. There were a number of barriers to recommending acupuncture, including limited knowledge and cost burden to families.

## Introduction

Autism is a complex neurodevelopmental condition characterized by: (1) differences in social communication and social interaction and (2) restricted, repetitive behaviours, interests and activities.^1,2^ In addition to these core diagnostic features, people with autism often have associated delayed language, motor, cognitive or learning skills, difficulties with sleep, sensory or digestive issues, unusual mood or emotional reactions, focus and attention, and seizures.^3-14^ These challenges are lifelong and can have significant negative impacts on the children and their families.^15,16^

At present, there are no medications to the core features of autism.^17-22^ Associated self-injury/aggression or psychiatric challenges may be managed with medications,^17-22^ which may have adverse effects such as sedation, obesity, and dizziness.^17-22^ Preferred approaches to autism treatment are non-pharmacologic, including behavioural and developmental skills training, environmental adaptation, and parent education.^21,23^ However, these therapies require extensive time and resources.^24-26^

Complementary medicine refers to a “broad set of health care practices that are not part of that country’s own tradition or conventional medicine and are not fully integrated into the dominant health-care system”.^27^ Integrative medicine combines complementary and conventional medicine in a multimodal approach.^28^ It acknowledges patients’ beliefs, values and goals, considering each type of medicine to be equally important in promoting overall health.^29^ Traditional medicines are those indigenous to a specific culture. Collectively, this body of knowledge has been referred to as traditional, complementary and integrative medicine (TCIM), or complementary and integrative medicine (CIM).^30^

The use of complementary medicine in children and adolescents with autism is high (28% to 95%).^26,31^ Parents seek TCIM to address a wide range of health and functional issues, from core autism symptoms to improving attention, relaxation, managing digestive complaints, sleep and to promote general health.^32-34^ Reasons include limited conventional healthcare options, concerns of adverse effects of conventional medicines, and the perception that complementary medicines are “natural” and therefore safe.^25,26,32,33^

In the United States, parents most often seek information about complementary medicine from friends and family (35%), physicians (23%), and the internet (23%).^25,31,34^

Barriers to discussions with conventional healthcare providers include parents’ lack of comfort discussing complementary options, views of complementary medicine as being outside the scope of conventional health practitioners, and concerns about practitioners potentially disapproving of complementary medicine.^33,35,36^ Acupuncture is based in traditional Chinese medicine (TCM) with over 3000 years of history and is “the most common form of traditional medicine practice globally”.^37^ Specific points on the body are stimulated, often using fine needles, in order to achieve health benefits. There is a broad body of literature regarding the effectiveness of acupuncture for many conditions, including in children.^38-49^

Laser acupuncture uses a device that emits infrared light to stimulate acupuncture points, and arose from research on low level light therapy, or photobiomodulation, which uses non-thermal irradiance of photons to alter cellular activity.^50-52^ Laser acupuncture is relatively new, with approximately 50 years of history.^50,53,54^ It is considered a less invasive alternative to needle acupuncture due to its lack of skin penetration and minimal sensation.^55,56^

A recent systematic review of twenty-six studies involving healthcare professionals (mainly physicians and medical students) reported positive attitudes towards the integration of needle acupuncture into Western medicine.^57^ In this review, enablers to recommending needle acupuncture included prior positive experience, belief in the treatment’s efficacy, and patient demand. Barriers included lack of evidentiary knowledge or experience, exposure to the uses and timing of when to use acupuncture, resources such as time, providers, and funding, and paradigm differences.^57^ However, this review did not include use of acupuncture for children with autism. To our knowledge, there has not been research on healthcare practitioners’ views on the use of acupuncture, including laser acupuncture, in the treatment of core features or co-occurring symptoms/functional deficits associated with autism. Given the interest in complementary therapies for children with autism, an increased understanding of healthcare providers’ knowledge and perspectives of acupuncture, including laser acupuncture, is important.

## Method

Ethics approval was obtained from the University of Alberta (Pro00131432). The authors declare no potential conflicts of interest with respect to the research, authorship, and/or publication of this article.

This interpretive qualitative description study aimed to explore healthcare providers’ views of acupuncture, including laser acupuncture, for children with autism. An interpretive description qualitative research approach is often used to guide qualitative inquiry in applied settings with the goal of informing clinical practice.^58^ Exploring these views will help us understand the factors contributing to decisions to recommend or not recommend acupuncture for children with autism. Specifically, our study sought to answer the following questions: (1) how do healthcare providers perceive acupuncture as a therapy for children with autism? And (2) what are the reasons for healthcare providers to recommend or not recommend acupuncture for children with autism?

### Recruitment and Participant Information

We used purposive sampling common to qualitative research. We recruited by systematically reaching out to senior leadership in multiple programs and sites as well as to our existing networks to broadly access where children with ASD are seen. We sent a Letter of Initial Contact to the program managers who then dispersed it to their staff. We conducted interviews via video conference with individuals who expressed interest. As is true in all research, we only gathered data from those who consented to participate. We intentionally sought the perspective of people from a diverse group of professionals and did not limit it to those who had expressed interest in acupuncture. We are pleased to have acquired varied representation, with regards to discipline, work setting, type of work experience, and length of work experience.

Inclusion criteria were regulated healthcare providers who: (1) assessed and/or treated children with autism, (2) were in a position to recommend acupuncture, if they so desired, and (3) lived in Alberta, Canada. We sought diverse perspectives from those who had and had not recommended acupuncture. All participants provided informed consent verbally prior to the start of the interview.

### Data Collection

Author DD collected data through semi-structured individual interviews by Zoom. Verbal consent was obtained prior to the interview and DD introduced herself as an occupational therapist and acupuncturist with a private clinical practice that focuses on children with neurodevelopmental conditions. Interview questions are provided in Appendix 1. Interviews were audio recorded and transcribed verbatim using Otter.ai© software. Transcripts were cleaned and identifiers removed and replaced with codes, such as Psych 1, OT 1. The interviewer kept a reflexive journal throughout the process to document thoughts and reflections immediately after each interview, as well as personal assumptions and biases in relation to the content of the interview. These journal entries were utilized as part of the analytic process.

### Data Analysis

Data were coded and analysed using an inductive thematic analysis approach.^59^ Data were inputted into NVivo12 for organisation, coding and thematic development. First, the lead author immersed and familiarized herself in the transcribed data, reading and re-reading for meanings and patterns, and recording reflections, thoughts, and ideas for potential codes. Second, she performed data-driven coding, and collated codes into categories. Some data extracts were used more than once to fit into different categories. Categories were reworked though an iterative process into potential themes and sub-themes related to the objectives of the study. With co-author STH (senior author), these candidate themes were reviewed and refined over multiple meetings. Finally, themes were named, defined related to how they contributed to the objective, and summarized.

To ensure rigour and trustworthiness of the data analysis, the criteria of credibility, transferability, dependability and confirmability in qualitative research as outlined by Lincoln and Guba (2006)^60^ were used. As a practising OT/acupuncturist, the lead author acknowledges the preconceptions and prejudices inherently brought to interactions with research participants and in the analyses. Increased credibility was sought by gathering extensive material from a variety of healthcare professionals and triangulating data. STH supervised the coding and theme development to ensure the data were interpreted without prejudice.

## Results

Thirteen healthcare professionals who had worked with children with autism for 2 to over 30 years were interviewed (3 occupational therapists (OT), 4 speech-language therapists (SLP), 3 psychologists (Psych), 1 physiotherapist (PT), 1 child psychiatrist, and 1 developmental paediatrician) between July 2023 to January 2024. Interviews were 17 to 68 minutes long (average 60 min). Three participants worked in the school system, 2 of whom also had a private practice; 4 worked in a hospital setting; 6 worked solely in private practice. See Table 1 for respondents’ professional information.

**Table 1.** Respondents Professional Information

	Profession	Workplace setting	Years in practice
OT01	Occupational therapy	Edmonton public school board	29 years
OT02	Occupational therapy	Community	14 years
OT03	Occupational therapy	Community	32 years
SLP01	Speech-language pathology	Hospital	8 years
SLP02	Speech-language pathology	Edmonton public school board and community	18 years
SLP03	Speech-language pathology	Pre-school and community	33 years
SLP04	Speech-language pathology	Hospital	9 years
PT01	Physiotherapy	Community	20 years
Psych01	Psychology	Community	2 years
Psych02	Psychology	Community	14 years
Psych03	Psychology	Hospital	2 years
Paed01	Paediatrics	Hospital	28 years
Psychiatry01	Child psychiatry	Community	35 years

None of the participants were practitioners of acupuncture. After 13 interviews, once interviews no longer provided novel themes, the team agreed that data had reached saturation and we stopped further recruitment.

Analysis of data identified 4 themes.

### Theme 1. “Interesting, Tell Me More”: A Lack of Familiarity, But Interest, in Acupuncture for Children With Autism

All participants (n = 13) had heard of needle acupuncture and most (N = 10) had experienced acupuncture for themselves or a family member for a wide range of uses, including pain, headaches, physical injuries, sleep, infertility, and stress. Most participants reflected positively on their personal experiences, except for 1 participant who reported that the needles exacerbated the pain of her sprained ankle. This individual also expressed the most apprehension about needle acupuncture. There was general understanding that acupuncture involves needling along meridians. For example, participants elaborated that acupuncture “keeps all the systems open and together and flowing so the whole body works as a healthy system” (SLP 02), and uses “key points to facilitate movement of energy within the body” (OT 03). An SLP and a psychologist both believed that “there’s some evidence behind it” but they, like all other participants, acknowledged they knew very little about acupuncture.

Overall, our participants were mostly unaware of acupuncture for children with autism. Four participants (2 OTs, psychiatrist, developmental paediatrician) had children in their practice who received acupuncture, but they did not know the specifics of what was being treated or whether needle or laser was being used. One participant shared her perspective of 1 young patient on her caseload. “He was very much helped by acupuncture. ….I think it helped calm his autonomic nervous system, it calmed the stress reactivity in the body” (Psychiatry 01).

Despite their lack of knowledge, most participants were interested in learning more about acupuncture for children with autism, including an SLP who commented, “Until you got a hold of me, I didn’t know it was a thing. So, I am interested to hear more. Very interested” (SLP 02), and a psychologist, who indicated her interest:

I’m very open to it (acupuncture). I’d like to see that there’s some research behind it…and there’s actually some benefit in the studies and there’s some scientific input …but yeah, I'm very open to it. I think that if it’s beneficial, why not try it? (Psych 01)

All participants indicated that there was little to no discussion of acupuncture for children with autism between colleagues, in their workplace, or in the professional journals they read. Their knowledge of acupuncture was based mainly on their personal experiences. None had referred a child with autism for acupuncture.

### Theme 2. “I Think We Need to Explore All Avenues”: Openness to Integrative Practice for a Complex Condition

Despite overall low familiarity with acupuncture for children with autism, there was openness to its use. For example, 1 PT explained:

I think we need to explore all avenues. And sometimes there are things that we can't necessarily concretely explain. I don't think we can limit families, clinicians, children from having access to something that may actually be significantly of value to them. (PT 01)

Participants acknowledged that a one-size-fits-all approach to intervention does not work with children with autism, as indicated by a comment from 1 occupational therapist: “I think it’s an excellent idea to be looking further into how we can help these children with autism because they’re lovely and they have so much to offer, but sometimes we just can’t reach them” (OT 01). Another also noted that there are limitations to current therapies: “There’s so many of them (children with autism) who are over-medicated and it’s not affecting the difficulties they’re having” (OT 02). As such, professionals were keen to learn about strategies that could enhance support for children and families. The potential value of acupuncture was related to 2 areas and reflected the complexity of autism: perceived gaps in conventional therapies (subtheme 1), and considerations related to potential healthcare induced trauma (subtheme 2).

#### Subtheme 1: The Potential Value of Acupuncture for Whole Person Health

Participants discussed the potential value of incorporating acupuncture as 1 component of comprehensive therapy to help address perceived gaps in conventional therapies. For example, an OT felt that stress and anxiety were not adequately addressed using mainstream occupational therapy strategies. She felt that acupuncture could be beneficial if it could help to decrease stress and anxiety:

We sort of go down this path that somehow we can use the sensory system to support the child in their development in a broad way. I don’t think there’s a lot of success with that….if acupuncture can help those kids resolve some of that stress and anxiety, it gives an opening to allow for other interventions… developmental interventions to support them to gain or regain skills that they have had. But, in the absence of that [decreasing stress and anxiety], I think it’s really difficult. (OT 03)

Both physicians unequivocally did not believe acupuncture could help the core features of autism, but they and many other participants wondered if it could benefit co-occurring physical and mental conditions and regulate the nervous system. For example, the PT related:

I think it's excellent that there's other things being looked at that potentially could be helping them, but I don't know enough about acupuncture to know specifically how that could be helping them. I do know that as long as it helps to calm their body and to regulate their body, I think that's excellent. (PT 01)

Participants used the term ‘holistic’ when discussing their views on acupuncture:

If you look at the DSM, a lot of the symptoms describe our autonomic nervous system reactivity. A word like agitation…anxiety, it's got underlying states of arousal in the nervous system. I wonder if in more holistic practices like acupuncture, whether there's more of an appreciation for this, whereas in western medicine, there is not. (Psychiatry 01)

The psychiatrist felt strongly that the key to helping children with autism was to manage toxic stress, as a child’s brain is better able to adapt and grow when their sympathetic and parasympathetic nervous systems are in balance. One psychologist also reflected on the mind-body connection, reinforcing that she often considers the whole person: “It’s not just your brain right, like, your whole body is connected…so I think acupuncture ties into that” (Psych 01).

Participants also consider influences on behaviour that might be considered outside of a neurological basis of autism. An OT expressed a need for clinicians to look at autism differently, ”supporting, for example, the nervous system that can then, right from the root, help create gains in all other areas” (OT 02). She went on to add how allergies, intolerances, pain, and sleep can affect a child’s regulation, mood and irritability. Finally, she suggested to:

figure out how we can look at the big picture and…explore different interventions that can assist overall - and/or pieces of - so when something, you know, the traditional sort of interventions are not working, then how can we think outside the box and explore all other ones and continue to build evidence. (OT 02)

#### Subtheme 2: Laser Acupuncture May Help Address Concerns about Healthcare Induced Trauma

Participants expressed concerns that many of their clients had prior traumatic healthcare experiences and/or were afraid of needles, and that acupuncture could be a source of physical or emotional trauma for children with autism due to needle phobia, anxiety, cognitive rigidity, and/or tactile sensitivity. Two clinicians stated that needles were a reason they themselves had never tried acupuncture.

Participants preferred that potential acupuncture practitioners would be experienced with children with autism. “I don’t want to traumatize the kid to give them this treatment. I want the treatment to work for the kid. I don’t want the kid to work for the treatment” (Psych03). Participants felt that there was an additional layer of vulnerability due to communication differences in children with autism: “I am quite tentative to be hands-on with children with autism that cannot say “No” aside from pulling away or biting us or whatever” (PT 01).

Participants had not previously heard of laser acupuncture, and were generally interested that needleless acupuncture using a laser was an option. After an explanation of the device and its administration, all participants had a more positive view of lasers compared to needles for acupuncture. Most welcomed the laser as a less intrusive instrument, “I can imagine them finding it interesting rather than being fearful if they’ve already got an experience of needle fear” (SLP 01), and “This sounds like something that is low risk and they might benefit from it…might as well give it a try” (Psych 01).

### Theme 3. “The evidence I Need Is Change in a Child”: Both Empirical Evidence and Clinical Experience Are Important to Inform Clinical Decisions

While participants expressed the need for peer reviewed evidence and formal learning opportunities (subtheme 1), they were also receptive to other forms of knowledge like word of mouth and anecdotal evidence (subtheme 2). Participants wanted more knowledge about acupuncture, including laser acupuncture, to feel confident in making informed decisions and supporting families to do the same.

I feel like there’s been a shift between physicians acting in a very paternalistic way and being negative and discouraging to kind of respecting parents’ ability to make decisions about their kids…empowering them and supporting them in making informed decisions. (Paed 01)

In addition to knowing more about how acupuncture works and what it might be beneficial for, they wanted evidence supporting its use. Participants were clear that different types of evidence informed their clinical decisions.

#### Subtheme 1: Peer Reviewed Evidence Is Preferred and Ideal to Inform Treatment Decisions

Across all professions, clinicians stated they required peer reviewed evidence, in the form of well-designed clinical trials and systematic reviews, to be comfortable recommending acupuncture. Without this knowledge, many participants did not feel confident or competent to make the recommendation, and they “would have to see the evidence before forming an opinion and making any recommendations…[and] evaluate the quality of the evidence” (Paed 01). However, busy practitioners faced a lack of time to search for and read journal articles. As such, clinicians wanted this information in digestible ways:

I think that’s where having something that was succinct and trustworthy, that the evidence doesn’t have to meet some unattainable goal, but at least to have it distilled in a trustworthy way so that there is transparency to what we’re actually trying to decide. (Paed 01)

In addition to evidence on effectiveness, participants reinforced that, prior to recommending acupuncture, they would require clarification on how it could help, indications for its use, potential risks, and how to manage needle phobia. For example, a psychologist reinforced the need to consider harm within evidence-based decisions:

I’m a pretty scientific based person, so if I can find some peer reviewed, good quality journals that say, you know, this is beneficial ... the benefit outweighs the harm, then that would be something that I would find credible. (Psych 01)

Participants did talk about scepticism towards TCIM within the conventional healthcare community. This mistrust was driven by the perception that complementary and integrative practices are less well researched, and that unregulated products or services may be marketed towards vulnerable parents desperate to find a cure for their children:

There are some physicians whom I think would be quite sceptical of the broader range of integrative treatments; I think that it’s against the background of a lot of commercially available treatments that are poorly researched that are kind of marketed and I think there’s a sense that people take advantage of families of kids on the autism spectrum. (Paed 01)

One therapist was concerned that families use acupuncture in lieu of conventionally accepted therapies or medically indicated interventions, “I would be very hesitant, if not in opposition, to somebody suggesting acupuncture as a replacement for speech and language therapy if there were communication concerns” (SLP 01). However, she voiced that acupuncture could be used as an adjunct intervention.

#### Subtheme 2: Clients and Colleagues Can Also Be Trustworthy Sources of Evidence

Non-research-based evidence was also considered valuable, especially clinical experience. Word of mouth from clients’ personal experiences was important:

[If I] “heard from families who have tried it, has a really positive result, right, and positive outcomes…I would be more than willing to pass that information along with the caveat that…I don’t know a lot about it, but this is what I’ve heard from people…” (SLP 01).

Participants also noted acupuncture’s long history, “They’ve been around for thousands of years, so obviously, if people are still practising them, then there’s benefit to them” (Psych 01). Participants appreciated opportunities to learn about acupuncture through discussion in collaborative groups or professional presentations:

Certainly, somebody coming in and talking would be a good start, if there were studies, but again, I’m a little sceptical about [using] studies just because the disorder is based on a bunch of behaviours, and we don’t know if what’s going to work on one child will work on another....So I think mostly hearing from somebody about what it might do and why. (SLP 03)

Participants noted that the treatment of children with autism is complex and nuanced. Observing an acupuncture treatment could make them feel more confident in recommending it to families, “I want to make sure I am well educated in areas and I think shadowing someone else in action [would be helpful]” (PT 01). Several participants also felt that collaborating with an acupuncturist would support their knowledge base. “I would have loved to be able to work with the people that were working with the individuals during this so that we could work together because I think that would be very helpful” (OT 01).

Trust in the source was very important regarding anecdotal evidence, “I guess it depends where it’s coming from, right. You’re gonna place different weight on people’s opinions and experiences” (SLP 01). Two therapists weighed the importance of clinical experience as an important source of evidence: “Somebody who’s actually working as opposed to just pure research” (SLP 03) while another said, “The evidence I need is change in a child” (OT 03).

### Theme 4. Practical Considerations for Recommending Acupuncture

Aside from needing more information, there were practical barriers to recommending acupuncture therapy, including (lack of) knowledge of skilled acupuncture practitioners, cost and scope of practice.

Participants were interested in a skilled clinician who understands autism and, aligned with their focus on physical and emotional safety, knows how to treat children without causing health related trauma. When asked what may prevent the respondent from recommending laser acupuncture, SLP 03 replied, “It would be just lack of knowledge about the procedure and lack of awareness of skilled practitioners.” Not knowing providers who understood autism was a barrier to recommending acupuncture. “I don’t know all the providers out there…I think there are some more skilled and less skilled…and there are providers that have more understanding of autism and how to engage an individual with autism” (Psychiatry 01).

Cost was perceived as a significant barrier to recommending acupuncture, especially if it was not covered by the public healthcare system: “Services from OTs and other health professionals are not necessarily publicly funded, so that adds a layer…sort of, out of pocket cost for families” (Paed 01). Participants expressed a need to protect a population that is, “highly vulnerable and there’s a lot of emotion and hope” (OT 03), and that parents, as they search for ways to help their child, “aren’t being taken advantage of, like, financially” (SLP 01).

Although participants were supportive of acupuncture, and especially laser acupuncture, for children with autism, they also expressed uncertainty about whether recommending acupuncture was within their scope of practice and aligned with their obligations to their professional Colleges: “It’s not within my area of competency to recommend, but in terms of my own openness to any of this, I’m very open” (Psych 02), and “It’s really not in my scope of practice to prescribe it, but I feel comfortable having that conversation and supporting the family if they’re thinking about it” (Psychiatry 01). In contrast, the regulatory college for physiotherapy recognizes acupuncture as a restricted activity so the physiotherapist was very comfortable with this modality. The paediatrician pondered whether it was within the physician’s scope of practice and responsibility to recommend the “full continuum of what’s out there, or should some of this information be shared more directly with the parent community” (Paed 01).

## Discussion

Our findings suggest that healthcare professionals across multiple disciplines were unaware of acupuncture, including laser acupuncture, for children with autism. However, the attitudes of our participants towards using acupuncture for this population were positive and open to formal and informal forms of evidence regarding its effectiveness and safety. This is consistent with previous surveys of nurses’ and physicians’ receptivity of acupuncture for various conditions.^57,61-63^

Importantly, our study includes the perspective of a diverse group of health professionals. Overall, there did not appear to be a significant qualitative difference in the views expressed by physicians and allied health professionals. Familiarity with acupuncture, whether from personal experience or professionally (children on their caseload) seemed to influence their perspectives.

The mechanisms of action of acupuncture are not fully known,^57,64-68^ and there is continued scepticism of its efficacy amongst some healthcare practitioners.^69^ Although preliminary evidence on the efficacy of acupuncture for children with autism exists, findings are mixed at this time. Two earlier systematic reviews (published in 2011)^70,71^ concluded there was no to limited evidence supporting acupuncture for autism while 4 more recent ones (published between 2018 and 2023) cautiously suggest that acupuncture may be beneficial for autism.^72-75^ However, the quality of evidence is low or very low, given issues of poor methodology, small studies, and high risk of bias.^72-75^ Furthermore, only 2 small trials have investigated laser acupuncture for ASD.^76,77^

Some evidence of benefit exists (adult and pediatric literature) for several co-occurring conditions that are common in children with autism, including insomnia, pain, anxiety, depression, and digestive issues.^38,40,44,45,78-84^ These conditions can adversely affect social, emotional, psychological and developmental outcomes.^3,85-90^ The physicians and many of the therapists were interested in acupuncture for these co-occurring conditions.

Physician interest in acupuncture is frequently motivated by lack of response to conventional treatments, patient interest, prevention of adverse drug reactions, a search for a different paradigm to explain the health condition, and/or interest in treating the whole person (physical, emotional, mental, social and spiritual dimensions).^91,92^ Our participants acknowledged that current therapies may not be able to fully meet the needs of many children with autism, and were interested that acupuncture may help address this gap.

Children with autism can be more vulnerable to healthcare trauma for many reasons.^93-96^ While needle acupuncture is considered safe and well tolerated for children when provided by trained practitioners,^97^ laser acupuncture was of particular interest to our participants for its less invasive and non-penetrating nature.^55,56^ Finding acupuncture providers experienced in providing autism-sensitive service was felt to be as important as avoiding needles and healthcare trauma. This is particularly important to meet the needs of the diverse behaviours, capabilities, fears, and cognitive abilities among children with autism.

## Limitations

There are a number of limitations to this study. As this was a qualitative investigation, primary and secondary outcomes were not established *a priori*. As a result, deeper exploration of some issues that emerged during the interviews did not occur and could be a focus of future studies. For instance, children with autism exhibit substantial functional and phenotypic variability (eg, non-verbal, fearful, or tactile sensitivity) which may affect the feasibility of acupuncture and choices on how to stimulate acupoints (eg, needle, laser). In another instance, physicians wondered whether acupuncture was within their scope of practice to recommend. Since any therapy that is known to be effective can be considered within a physician’s scope to recommend, it would have been valuable to clarify what “scope”, in this context, represented to the physicians in our study, and how awareness of strong evidence and clinical guidelines supporting specific indications for acupuncture, such as pain, may have impacted their responses.

Other members of the broader multidisciplinary team, like behavioural consultants and teachers, were not included. Although they do not make healthcare recommendations or referrals, they may have unique perspectives on acupuncture for children with autism. Parents, as decision-makers for their child and consumers of complementary therapies, were also not included.

The participants in our study represented diverse disciplines, practice settings and clinical experience as these factors may influence perceptions of acupuncture. We emphasized to our participants that the primary focus of our study was to understand their perspectives - whether positive, negative, or neutral - on acupuncture for children with autism. Despite this, there were no strong dissenting views. It is possible that people who agreed to be interviewed by a paediatric occupational therapist/acupuncturist were predisposed to view this topic favourably and with curiosity and interest. Only 1 participant expressed reservations about acupuncture, but she was open to its potential as a complementary therapy. Our findings therefore, may not be generalizable to all healthcare professionals who work with children with autism. The extent to which participants’ professional backgrounds, clinical experience and work settings may have influenced their views was not explored. This could be investigated in future studies.

There was inconsistency in some of the interview questions, which asked about acupuncture for “children with autism” and acupuncture “for this condition” (ie, autism). Although this ambiguity may have influenced responses, participants across the disciplines were consistent in stating their doubts that acupuncture could treat core autism symptoms (“acupuncture for autism”), while otherwise remaining open to its use (“acupuncture for children with autism”) for a wide range of issues encountered in clinical practice with children with autism, like pain, sleep, gastrointestinal symptoms, mood, all of which can affect function and behaviour.

Lastly, we did not explore reasons for lack of awareness about acupuncture. Future research could explore reasons for the lack of awareness, such as lack of high quality empirical data or limited exposure to acupuncture and other complementary integrative medicine practices during formal training at university.^67^ Future research could also explore their views for acupuncture for co-occurring conditions often experienced by children with autism that may have more evidence (eg, pain, anxiety).

Future qualitative research should explore views about acupuncture of parents as well as other members of the non-healthcare team involved in the care of children, such as social workers, teachers, education assistants and applied behaviour analysts. This would be helpful to inform clinical care, as would the views of acupuncturists, including those who use lasers and who have been involved in the care of children with autism. Our participants acknowledged the limitations of conventional therapies. Further high quality research on the efficacy, safety, feasibility and acceptability of acupuncture, especially laser acupuncture, in the management of autism are of interest. Growing evidence could foster greater awareness and improved care.

## Conclusion

Acupuncture is a modality that many healthcare professionals working with children with autism have not considered recommending. Despite this, the attitudes expressed by our participants were generally positive. Responses suggested curiosity and openness about exploring the use, evidence and potential of acupuncture to help meet gaps not currently met by conventional therapies. Participants felt they did not have enough knowledge to make informed decisions.

## Appendix

### Interview Guide

#### Topic 1: Background Questions

1. What is your name, position and your role in this clinic/hospital?
2. Do you work with children with autism, and in what capacity?
3. How much of your caseload is with this population?
4. How long have you worked as a therapist/physician?
5. What do you know/heard about acupuncture in general?
6. What do you know/heard about acupuncture for children with autism?

#### Topic 2: Acupuncture and Autism Spectrum Disorder

1. What do you think of acupuncture for children with autism?
  a. What are the benefits, if any?
  b. What are the harms, if any?
2. Have you ever recommended acupuncture for children with autism?
3. What might prevent you from recommending acupuncture for this condition?
4. What would help you recommend acupuncture for this condition?
5. What evidence would you need for you to feel comfortable recommending this therapy? Why?

#### Topic 3: Laser Acupuncture

Repeat Topic 2 questions specific to laser acupuncture.
